# Smart gene therapeutics for selective targeting of myofibroblasts derived from hepatic stellate cells and limited expression under inflamed conditions

**DOI:** 10.1002/ctm2.991

**Published:** 2022-08-02

**Authors:** Dodam Moon, Hyomin Park, Injoo Hwang, Areum Cha, Hyunji Yun, Jaewon Lee, Sung‐Hye Park, Eun Ju Lee, Hyo‐Soo Kim

**Affiliations:** ^1^ Department of Molecular Medicine and Biopharmaceutical Sciences Graduate School of Convergence Science and Technology Seoul National University Seoul Republic of Korea; ^2^ Biomedical Research Institute Seoul National University Hospital Seoul Republic of Korea; ^3^ Interdisciplinary Program in Stem Cell Biology Seoul National University of Medicine Seoul Republic of Korea; ^4^ Department of Pathology Seoul National University College of Medicine Seoul Republic of Korea

Dear Editor,

Recently, we reported the role of transcriptional intermediary factor‐1γ (TIF1γ) from hepatic stellate cells (HSCs) in preventing liver fibrosis.^1^ Therefore, an effective strategy to supply *TIF1γ* during liver injury offers significant therapeutic promise. Here, we devised a strategy to induce *TIF1γ* expression selectively in HSCs exclusively under inflamed liver conditions. We developed a TGFβ1‐promoter‐driven construct that induces *TIF1γ* expression in an inflamed liver undergoing fibrosis.^2^, ^3^ Additionally, we used a liposome–vitamin A (LiVitA) conjugate as a vehicle because vitamin A is selectively taken up and stored by HSCs in the human body.^4^

To evaluate the potential of *TIF1γ* in managing liver fibrosis, we systemically injected a cytomegalovirus (CMV)‐driven plasmid (pCMV‐mTIF1γ) into mice with thioacetamide (TAA)‐induced liver injury. Consequently, the area of collagen deposition in the injured liver was significantly reduced (Figure 1A). We then checked human specimens to test the applicability of TIF1γ as a therapeutic agent and of the TGFβ1‐promoter as the smart switch that should be turned on only in inflamed or injured liver. Cirrhotic human liver showed no expression of TIF1γ and very strong and wide expression of TGFβ1 and the fibrosis marker αSMA, which was in direct contrast to the pattern observed in normal human liver (Figure 1B). Staining with the HSC‐marker CRBP1 demonstrated that HSCs expressed TGFβ1 (yellow circle) and αSMA (yellow arrow) in the cirrhotic liver (Figures 1B and S1).

FIGURE 1Transcriptional intermediary factor‐1γ (*TIF1γ*), anti‐fibrosis gene in liver fibrosis. (A) Fibrosis staining and quantification of collagen deposition in mouse liver. Experimental schema for systemic injection of mTIF1γ plasmid vector (red arrow) into mice with liver injury induced by thioacetamide (TAA) administration (blue arrow). Quantification of liver fibrosis using picro‐sirius red staining in three groups (.83% ± .29% in control vs. 3.08% ± .34% in TAA treatment vs. 1.08% ± .43% in TAA/pCMV‐mTIF1γ treatment). Each black box indicates an independent individual liver tissue. Mice, *n* = 3 in each group. Quantification of the fibrotic area is presented as the red portion (%) of the total area. Scale bar: 200 μm. (B) Immunofluorescence staining of human liver tissue. Staining of TIF1γ, αSMA, CRBP1 (marker for hepatic stellate cells [HSCs]) and TGFβ1 was performed in normal and cirrhotic human liver tissue samples. Cirrhotic human liver characterized by loss of TIF1γ, the abundant expression of TGFβ1 and the deposition of αSMA. The yellow arrow and circle indicate myofibrotic HSCs that expressed TGFβ1 and αSMA or TGFβ1 and loss of TIF1γ with CRBP1, respectively, in the cirrhotic human liver. Scale bar: 10 μm

**Figure:**
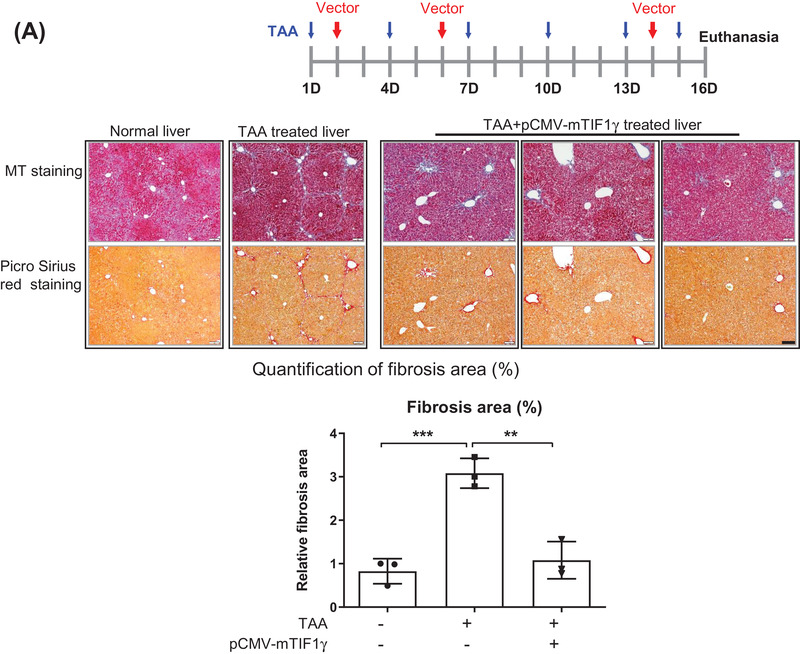

**Figure:**
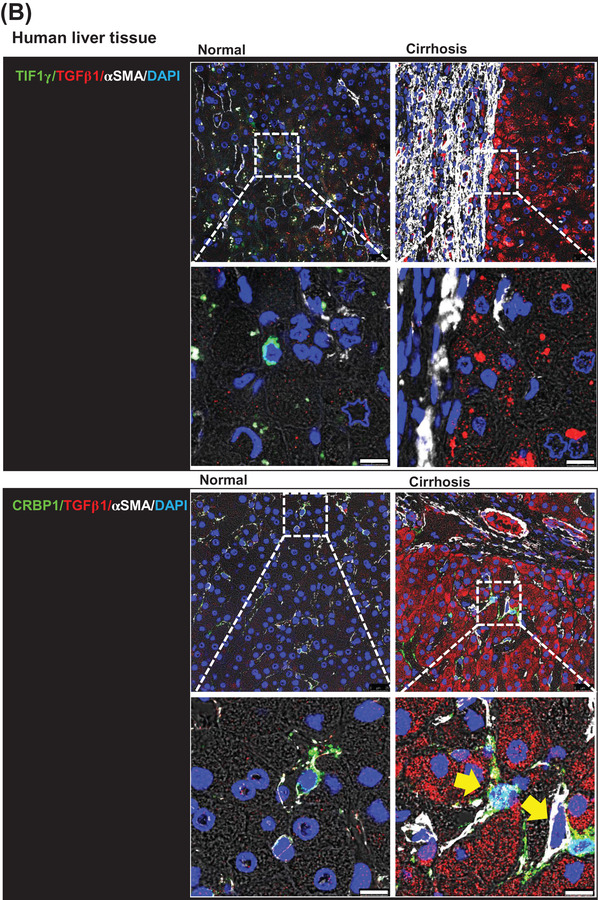

**Figure:**
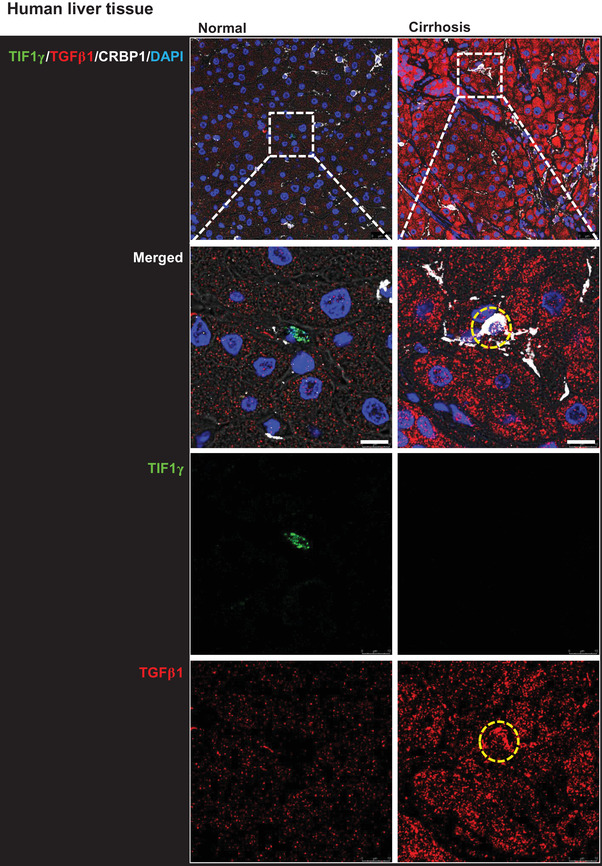

To enhance *TIF1γ* expression in HSCs under inflamed conditions with high TGFβ1 expression, we introduced the TGFβ1‐promoter‐driven *TIF1γ* into the human HSC line LX2 and assessed its selective expression according to the scheme shown in Figure 2A. The increased expression of αSMA or collagen type 1a (COL1A) under TGFβ1 was downregulated after transfecting the TGFβ1‐promoter‐driven *TIF1γ* (Figure 2B). We then verified the induction of TIF1γ expression under TGFβ1 upregulation, resulting in suppression of αSMA expression (Figure 2C).

FIGURE 2Construction and assessment of the switchable construct TGFβ1 promoter‐driven transcriptional intermediary factor‐1γ (*TIF1γ*) that selectively turned on in the presence of TGFβ1. (A) Schema showing the design of construct. Based on the positive feedback loop between TGFβ1 protein and its gene transcriptional activity, the TGFβ1 promoter‐driven TIF1γ will be turned on in the inflamed liver enriched with TGFβ1. (B and C) TGFβ1 promoter‐driven human TIF1γ turned on in the presence of TGFβ1 protein, leading to the expression of TIF1γ and suppression of fibrosis genes. Reverse transcription–quantitative polymerase chain reaction (RT‐qPCR) and the Western blot assay of LX2 cells. PPIA used for the normalization in RT‐qPCR. (D) TGFβ1 promoter‐driven mouse TIF1γ turned on in the presence of TGFβ1 protein at a similar level to cytomegalovirus (CMV) promoter‐driven mouse TIF1γ, leading to an expression of TIF1γ and suppression of fibrosis genes. RT‐qPCR results. PPIA used for normalization. (E) Immunofluorescent staining of TGFβ1‐treated three different LX2 cells (LX2 cells non‐transfected, transfected with pCMV‐emGFP or pCMV‐mTIF1γ/IRES‐emGFP) showing different expressions of TIF1γ and fibrosis genes. The construct pCMV‐mTIF1γ/IRES‐emGFP induces TIF1γ and suppresses αSMA in LX2 cells even in the presence of TGFβ1 (yellow asterisk). Scale bar: 25 μm in IgG and Non, 10 μm in others

**Figure:**
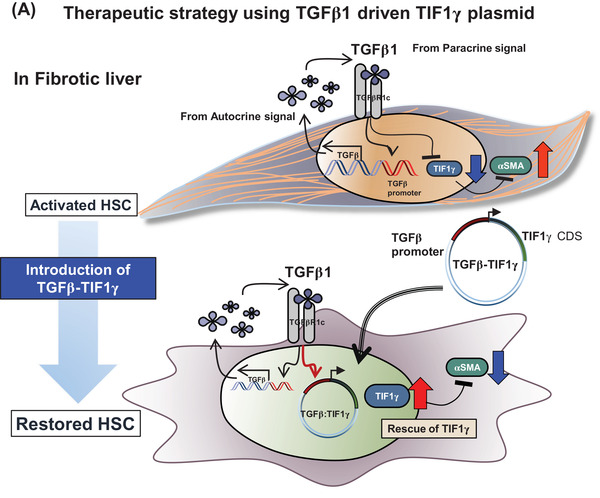

**Figure:**
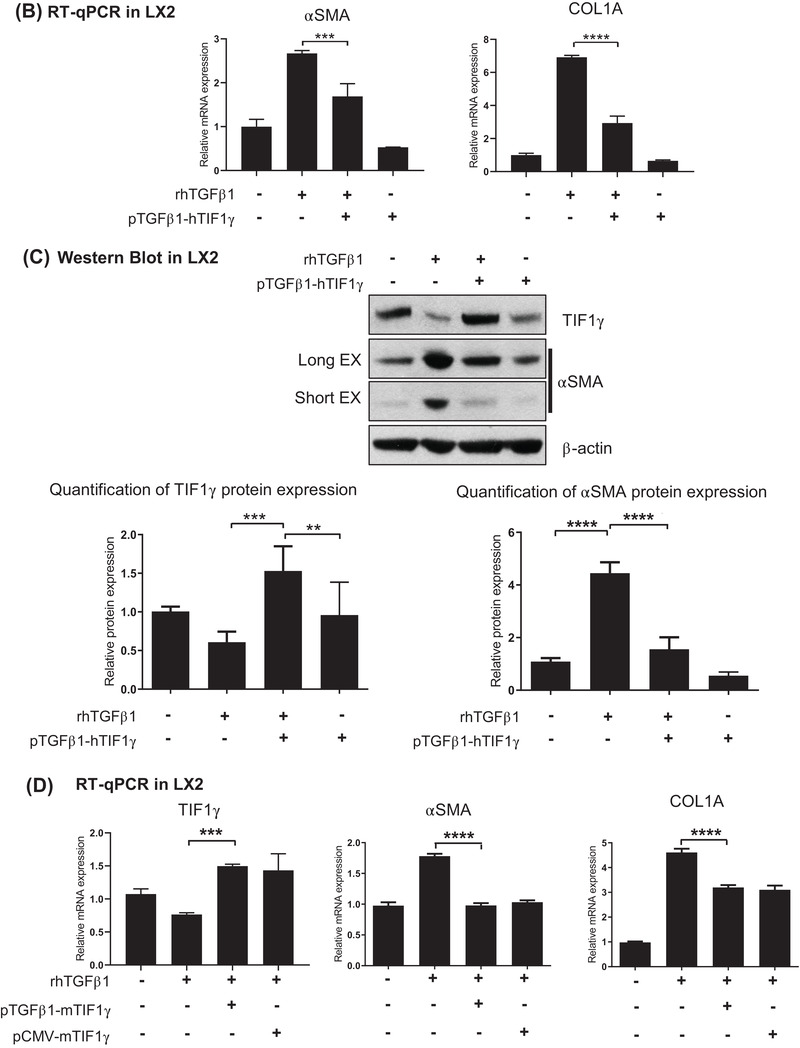

**Figure:**
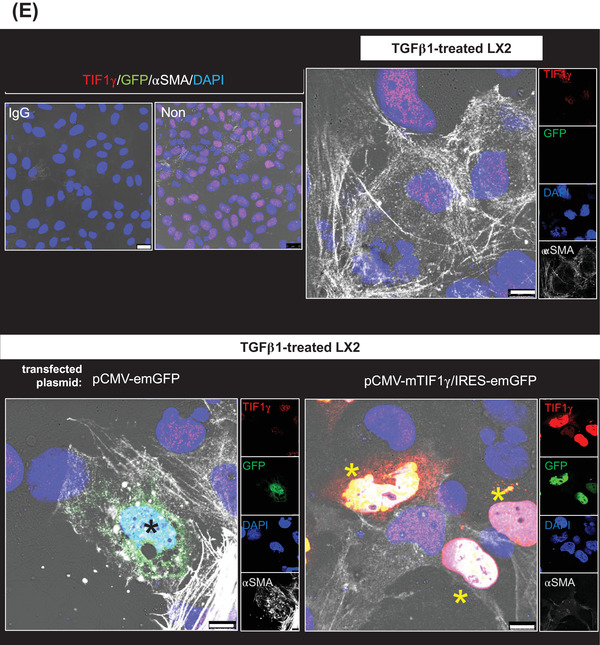

Because the homology of mTIF1γ and human TIF1γ (hTIF1γ) is 96% (Figure S2), we prepared mouse *TIF1γ* cDNA (mTIF1γ) for a TAA‐induced fibrotic mouse experiment and tested it in vitro using LX2 cells. CMV‐ or *TGFβ1*‐driven mTIF1γ reduced the expression of αSMA and COL1A in the human cells (Figure 2D). To observe the functionality of mTIF1γ at the single‐cell level, we transfected LX2 cells with the bi‐cistronic construct CMV promoter‐driven mTIF1γ/IRES‐driven emerald green fluorescent protein (emGFP) (CMV‐mTIF1γ/IRES‐emGFP). LX2 cells transfected with this construct expressed high levels of GFP and TIF1γ but did not express αSMA well (yellow asterisk, Figure 2E). By contrast, LX2 cells transfected only with GFP (pCMV‐emGFP) did not express TIF1γ but expressed αSMA (black asterisk, Figure 2E).

Vitamin A is stored mainly in HSCs in the body. Therefore, we prepared plasmid‐containing LiVitA (Figure 3A) and measured its size and zeta potential using transmission electron microscopy and dynamic light scattering, respectively (Figure 3B). Next, we prepared CMV‐emGFP plasmid‐containing LiVitA and administered it systemically via an intra‐cardiac injection to test whether the plasmid targeted HSCs selectively. emGFP was detected only in the liver (Figures 3C and S3). Subsequently, targeted delivery by CMV‐emGFP plasmid‐containing LiVitA was further demonstrated by fluorescence‐activated cell sorting analysis of isolated cells from liver, wherein approximately 4% of HSCs were transfected with GFP packaged in LiVitA, whereas only 1% of HSCs were transfected with GFP packaged in simple liposomes (Figures 3D and S4).

FIGURE 3The design and validation of gene therapeutics. (A) The design of the gene delivery system comprised a liposome–vitamin A conjugate (LiVitA) with retinol binding protein (RBP), which can target hepatic stellate cells (HSCs) that exclusively have receptors for RBP. (B) Zeta potential, size measurement and transmission electron microscopy (TEM) analysis of LiVitA with or without plasmid. The LiVitA and plasmid complex showed an average size of 161.6 ± 2.8 nm and a zeta potential of 44.1 ± 1.3 mV. With an increase in the components, the size of the complex increased (94.9 ± .4 nm and 96.2 ± 1.1 nm in the liposome and LiVitA, respectively), whereas the zeta potential decreased (56.3 ± 1.5 mV and 49.0 ± 1.8 mV in the liposome and LiVitA, respectively). TEM demonstrated that LiVitA and the LiVitA–plasmid complex were spherical with mean sizes of 96.2 and 161.6 nm, respectively. Scale bar: 500 nm. (C) In vivo evidence of selective targeting of HSCs. GFP detected only in HSCs of mouse liver but not in other organs after systemic infusion of cytomegalovirus (CMV)‐emGFP plasmid packaged in LiVitA. The immunofluorescent staining of mouse liver tissue. Scale bar: 25 μm in the upper panel and 10 μm in the bottom panel. (D) Schema of animal experiment where GFP gene packaged in LiVitA or simple liposome infused systemically into mouse with liver injury by thioacetamide (TAA) treatment. We harvested the liver and isolated single cells (hepatocytes and NPCs) from it. Bright‐field microscopy image and reverse transcription‐polymerase chain reaction (RT‐PCR) (Figure S4) showed the identity along with purity of the separated cells. In mice treated with LiVitA‐containing CMV‐emGFP plasmid, approximately 4% of HSCs in NPCs expressed GFP (4.17% ± .72%, *n* = 3), whereas more than 99.9% of hepatocytes did not express GFP. In control mice treated with simple liposome (without vitamin A) containing the CMV‐emGFP plasmid, approximately 1% of HSCs in NPCs expressed GFP (1.13% ± .51%, *n* = 3). Scale bar: 50 μm. (E) Polymerase chain reaction (PCR) of liver tissue genomic DNA (gDNA) from mice received systemic infusion of the pCMV‐emGFP or pTGFβ1‐tdTomato plasmid packaged in LiVitA. (F) Immunofluorescence of tdTomato in mouse liver. The *TGFβ1* promoter‐driven tdTomato turned on and left red tdTomato protein only in liver with TAA injury. Mice, *n* ≥ 5 in each group. Scale bar: 10 μm

**Figure:**
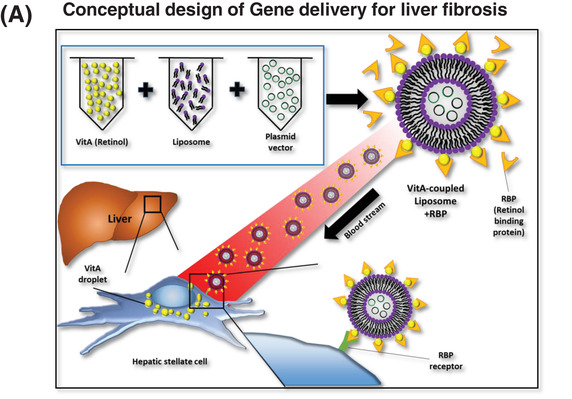

**Figure:**
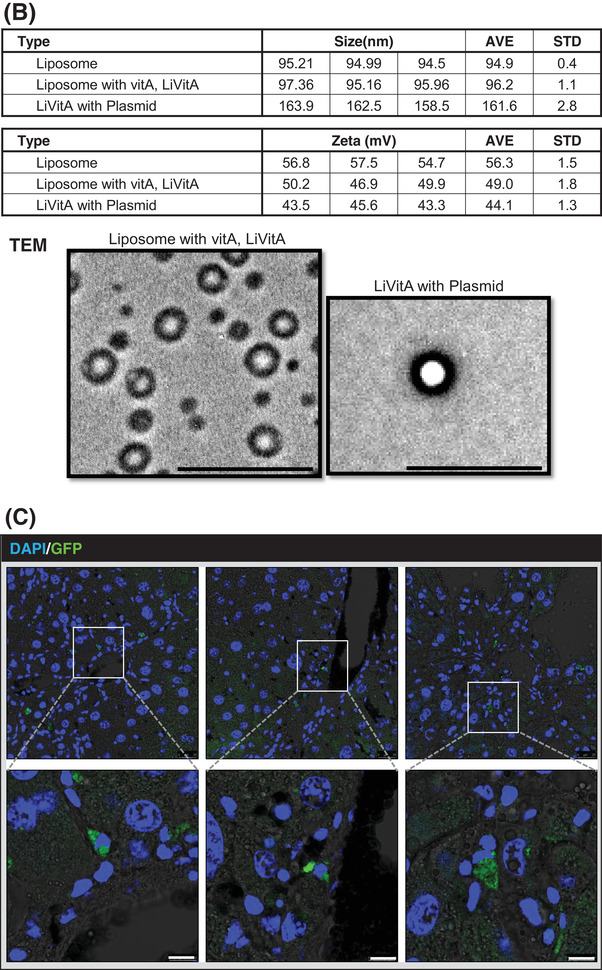

**Figure:**
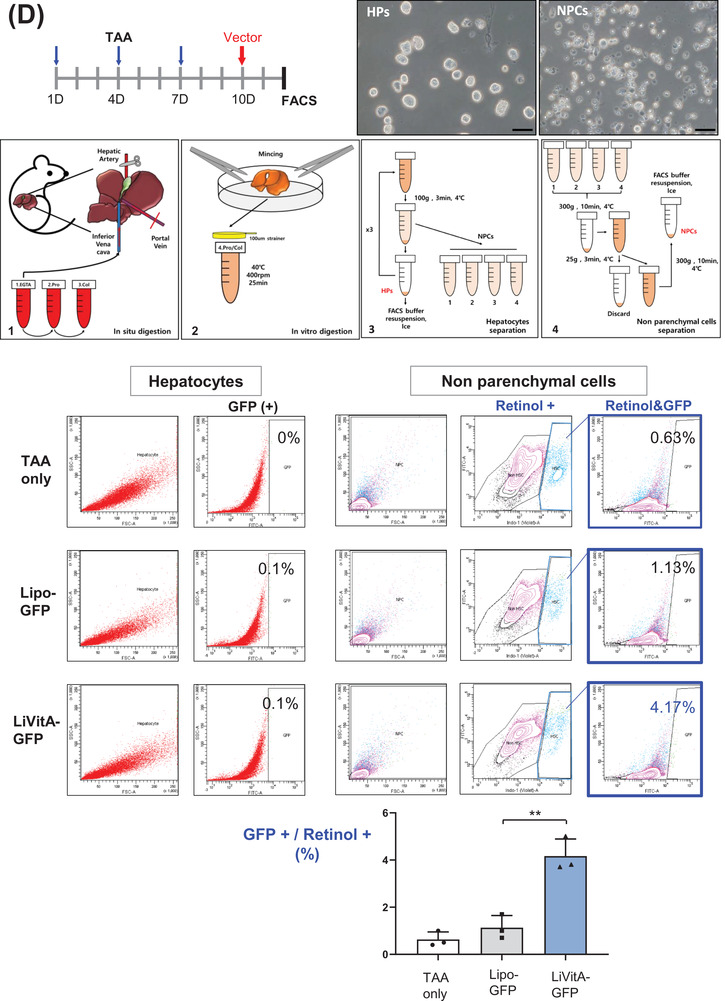

**Figure:**
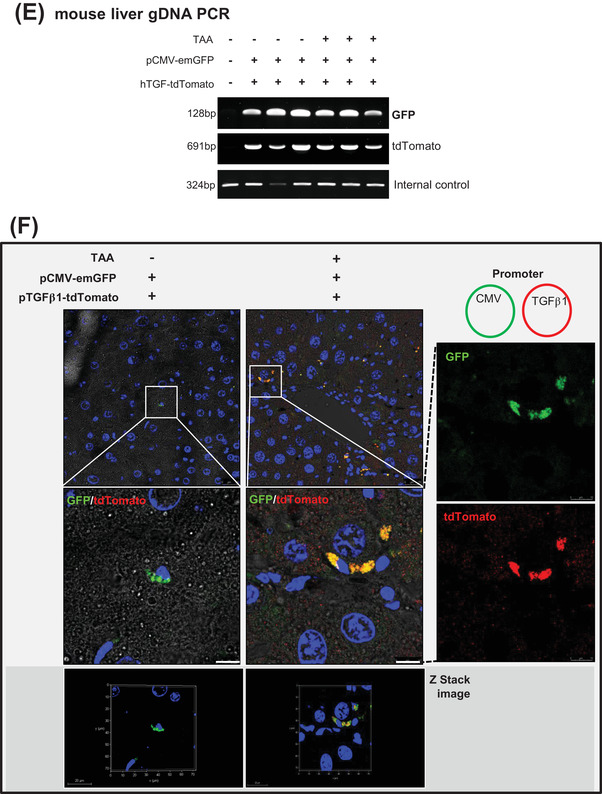

To assess the selective induction of the TGFβ1‐promoter under liver injury conditions in vivo, we compared constructs containing CMV promoter‐driven GFP versus TGFβ1‐promoter‐driven tdTomato. We verified plasmid delivery by performing polymerase chain reaction with the genomic DNA of liver (Figure 3E). The turn‐on selectivity of the TGFβ1‐promoter under fibrotic conditions was demonstrated using the immunofluorescence of tdTomato. TAA‐treated fibrotic liver showed the expression of GFP and tdTomato, whereas normal liver showed the expression of GFP only (Figure 3F).

We systemically administered LiVitAs containing TGFβ1‐promoter‐driven mTIF1γ plasmid four times via intra‐cardiac injections during liver injury induced by multiple TAA injections for 52 days. TGFβ1‐promoter‐driven TIF1γ gene therapy significantly reduced liver fibrosis as much as CMV promoter‐driven TIF1γ gene therapy did (Figure S5A,B). Moreover, its therapeutic effect was maintained even with a single injection (Figure S5C,D).

Next, we performed a codon‐optimization process by modifying the coding sequence of hTIF1γ^5^ and achieved remarkably enhanced expression of TIF1γ (Figure 4A). In the next in vivo animal experiment, we integrated this optimized sequence in the construct for future clinical application (Figure 4B). The mouse HSC line mSV40 was used to determine the functionality of the optimized hTIF1γ by assessing the downregulation of the genes associated with fibrosis, namely, *αSMA* and *COL1A* (Figure S6).

FIGURE 4Codon‐optimization of the construct to maximize the therapeutic efficacy. (A) Western blot assay of 293T cells transfected with the codon‐optimized construct. (B) Plasmid construction for clinical application. *TGFβ1* promoter‐driven optimized human TIF1γ (hTIF1γ) sub‐cloned into the pVAX1 plasmid. (C) Experimental schema of systemic injection of pTGFβ1‐optiTIF1γ/IRES‐tdTomato packaged in LiVitA once into mice with liver injury induced by thioacetamide (TAA) administration. Fibrosis staining and quantification in mouse liver. Quantification of the fibrotic area using picro‐sirius red staining is presented as the red portion (%) of the total area (normal [mice, *n* = 8], TAA [*n* = 3], pTGFβ1‐tdTomato [*n* = 8], pTGFβ1‐optiTIF1γ/IRES‐tdTomato [*n* = 9] and pCMV‐optiTIF1γ/IRES‐tdTomato plasmid [*n* = 6], 1.1% ± .3% in control vs. 10.2% ± 1.2% in TAA treatment vs. 9.5% ± 3% in TAA/Mock vector treatment vs. 4.8% ± 1.1% in TAA/pTGFβ1‐optihTIF1γ/IRES‐tdTomato treatment vs. 4.4% ± 1.4% in TAA/pCMV‐optihTIF1γ/IRES‐tdTomato treatment). Each black pattern indicates an independent individual mouse. Scale bar: 400 μm. (D) Aspartate aminotransferase (AST) and alanine aminotransferase (ALT) levels in mouse serum ([AST]: 53 ± 5.8 mU/ml in control vs. 657 ± 158 mU/ml in TAA treatment vs. 684 ± 194 mU/ml in TAA/Mock vector treatment vs. 492 ± 113 mU/ml in TAA/pTGFβ1‐optihTIF1γ/IRES‐tdTomato treatment vs. 570 ± 63 mU/ml in TAA/pCMV‐optihTIF1γ/IRES‐tdTomato treatment; [ALT]: 26 ± 4.1 mU/ml in control vs. 1508 ± 356 mU/ml in TAA treatment vs. 1268 ± 516 mU/ml in TAA/Mock vector treatment vs. 845 ± 208 mU/ml in TAA/pTGFβ1‐optihTIF1γ/IRES‐tdTomato treatment vs. 994 ± 136 mU/ml in TAA/pCMV‐optihTIF1γ/IRES‐tdTomato treatment). Each black pattern in the graph indicates an individual mouse. (E) Immunofluorescence of liver tissue. Gene therapy with pTGFβ1‐optihTIF1γ/IRES‐tdTomato suppressed liver fibrosis leaving downregulated αSMA in hepatic stellate cells (HSCs) positive for CRBP1 (yellow arrow). Abundant expressions of αSMA in fibrotic HSCs by TAA with pTGFβ1‐tdTomato were detected (white circle). Transfected cells with red tdTomato were positive for HSC marker, CRBP1. Scale bar: 25 μm in the upper panel and 8 μm in others

**Figure:**
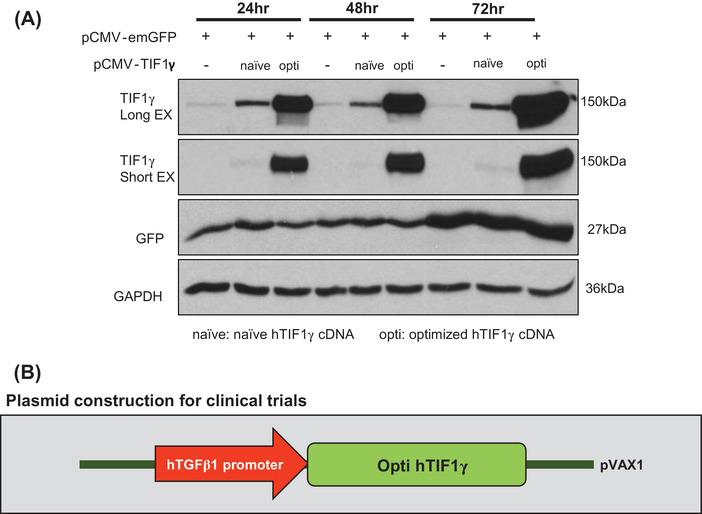

**Figure:**
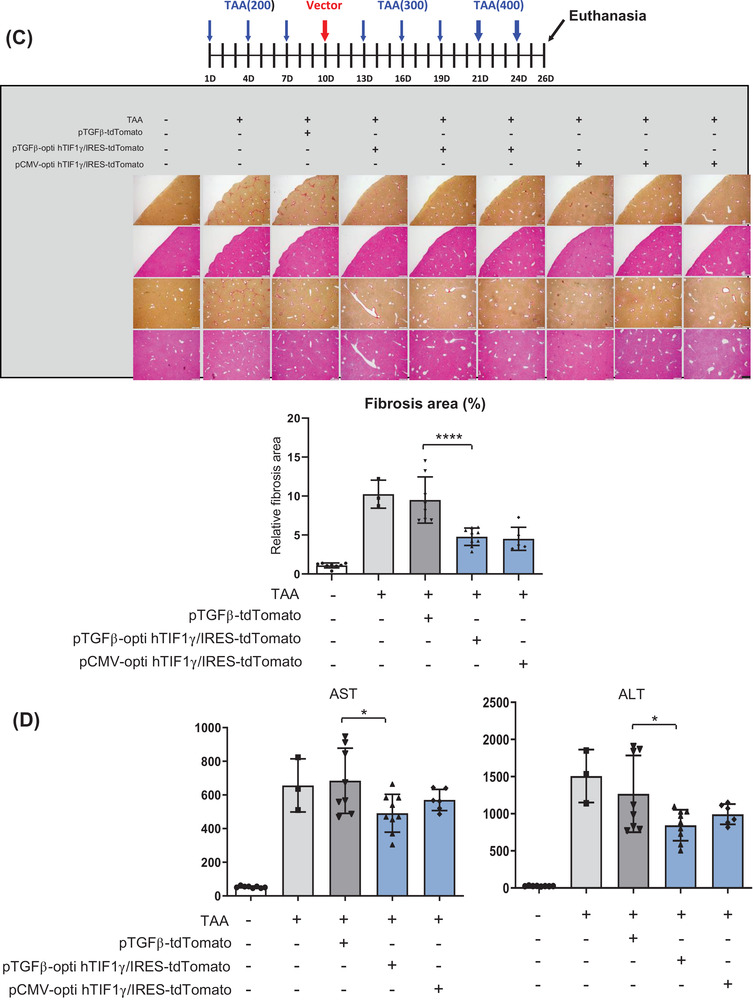

**Figure:**
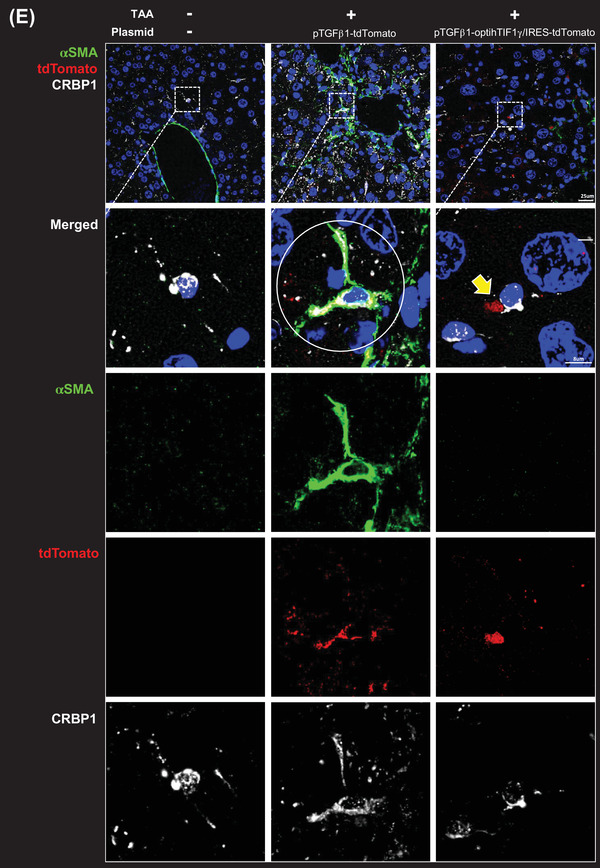

Then, we systemically administered LiVitAs containing TGFβ1‐promoter‐driven optimized hTIF1γ/IRES‐tdTomato in pVAX1 plasmid through a single intra‐cardiac injection to the liver injury mouse model stimulated with three rounds of triple TAA injections. Mild fibrotic transformation after one round of triple injection of TAA was observed on day 10, at which point we administered LiVitAs containing TGFβ1‐promoter‐driven optimized hTIF1γ/IRES‐tdTomato plasmid and evaluated the effects on day 26. The fibrotic areas with related genes and nonalcoholic fatty liver disease activity score (steatosis, inflammation and ballooning of hepatocytes) were significantly reduced by LiVitAs containing TGFβ1‐promoter‐driven optimized hTIF1γ plasmid, as well as by LiVitAs containing CMV promoter‐driven optimized hTIF1γ plasmid (Figures 4C and S7A–E). Serum AST and ALT levels were also significantly reduced (Figure 4D). As predicted, αSMA expression in HSC augmented by TAA was attenuated, and hepatocytes were restored to their original condition in animals treated with LiVitAs containing TGFβ1‐promoter‐driven optimized hTIF1γ/IRES‐tdTomato plasmid (Figures 4E and S7E). The selectivity and effect of LiVitAs containing TGFβ1‐promoter‐driven optimized hTIF1γ plasmid were proven by the CRBP1‐positive HSCs that express tdTomato but not αSMA, compared with the mock vector, pTGFβ1‐tdTomato.

In conclusion, we developed a smart strategy for gene therapy to specifically target liver injury and subsequent fibrosis; the strategy used a TGFβ1‐promoter‐driven optimized TIF1γ gene in liposome–vitamin A conjugate that enables the high expression of TIF1γ gene selectively in HSCs under the inflamed liver. The strategy of gene therapeutics has enormous potential for clinical application in patients with liver injury and subsequent fibrosis.^6^, ^7^, ^8^

## Supporting information

Supporting Information
