# Supplementary material for: Smart gene therapeutics for selective targeting of myofibroblasts derived from hepatic stellate cells and limited expression under inflamed conditions
Source: Clin Transl Med. 2022 Aug 2;12(8):e991. doi: 10.1002/ctm2.991 (PMC9345402; doi:10.1002/ctm2.991)
Supplement: Supplementary file 1 — Supporting Information [file CTM2-12-e991-s001.docx]

**Supporting Information**

**Smart Gene Therapeutics for Selective Targeting of Myofibroblasts Derived from Hepatic Stellate Cells and Limited Expression under Inflamed Conditions**

*Dodam Moon^1,3^, Hyomin Park^1,3^, Injoo Hwang^1,3^, Areum Cha^2,3^, Hyunji Yun^2,3^, Jaewon Lee^3^,*

*Sung-Hye Park^4^, Eun Ju Lee^2,3^*, Hyo-Soo Kim^1,2,3^**

^1^Department of Molecular Medicine and Biopharmaceutical Sciences, Graduate School of Convergence Science and Technology, Seoul National University, ^2^Interdisciplinary Program in Stem Cell Biology, Seoul National University of Medicine, ^3^Biomedical Research Institute, Seoul National University Hospital, ^4^Department of Pathology, Seoul National University College of Medicine, Seoul, Republic of Korea Seoul, Republic of Korea

***Corresponding Authors:**

Hyo-Soo Kim, MD, PhD

Department of Internal Medicine, Seoul National University Hospital

Molecular Medicine & Biopharmaceutical Sciences, Seoul National University

101 DeaHak-ro, JongRo-gu, Seoul 03080, Republic of Korea

Telephone: 82-2-2072-2226, Fax: 82-2-766-8904

E-mail: hyosoo@snu.ac.kr, [usahyosoo@gmail.com](mailto:usahyosoo@gmail.com)

ORCID ID 0000-0003-0847-5329

Eun Ju Lee, PhD

Biomedical Research Institute, Seoul National University Hospital

Interdisciplinary Program in Stem Cell Biology, Seoul National University of Medicine

101 DeaHak-ro, JongRo-gu, Seoul 03080, Republic of Korea

Telephone: 82-2-2072-1707, Fax: 82-2-2072-0398

E-mail: [leeunju@snu.ac.kr](mailto:leeunju@snu.ac.kr), [leeunju17@gmail.com](mailto:leeunju17@gmail.com)

ORCID ID 0000-0001-8489-271X

**ACKNOWLEDGEMENTS**

We thank Dr. Friedman for providing LX2 cells.

**FUNDING INFORMATION**

This research was supported by a grant of the Korea Health Technology R&D Project through the Korea Health Industry Development Institute (KHIDI), funded by the Ministry of Health & Welfare, Republic of Korea (grant number: HI14C1277). The funder had no role in the study design, data collection and analysis, decision to publish, or preparation of the manuscript.

**AUTHOR CONTRIBUTIONS**

Study concept and design: E.J. Lee and H. Kim. Acquisition, analysis, or interpretation of data: all authors. Drafting of the manuscript: E.J. Lee, D. Moon, and H. Kim.

Critical revision of the manuscript for important intellectual content: all authors. Funding acquisition: H. Kim. Technical support: E.J. Lee, D. Moon, H. Park, I. Hwang, A. Cha, H. Yun, J. Lee, and S. Park.

**CONFLICT OF INTEREST**

The authors declare no conflict of interest.

**SUPPORTING MATERIALS**

7 Supporting figures

Materials & Methods

**7 Supporting figures**


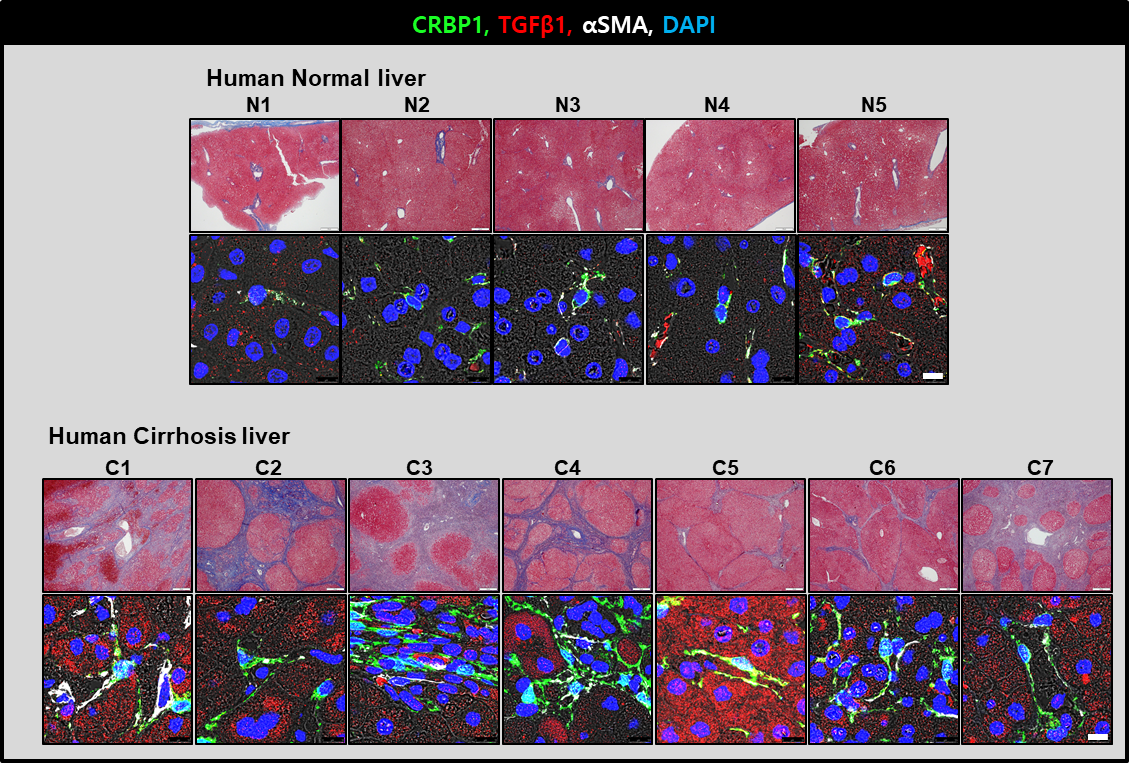


Figure S1. Immunofluorescence and Masson’s Trichrome staining of human liver tissue. Staining of αSMA, CRBP1, and TGFβ1 in normal and cirrhotic human liver tissues. Scale bar: 10 µm.


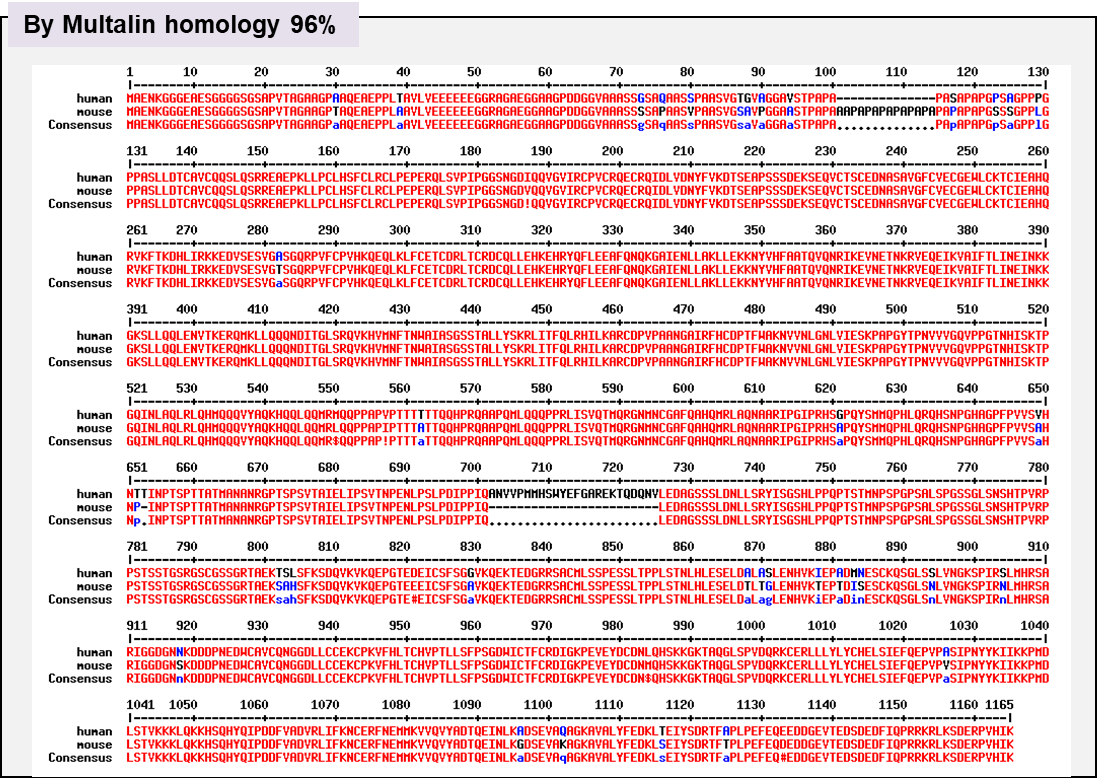


Figure S2. Alignment of human TIF1γ and mouse TIF1γ amino acids. The homology of mTIF1γ and hTIF1γ amino acids was analyzed using the Global Alignment BLAST tool of NCBI and Multalin Alignment tool.


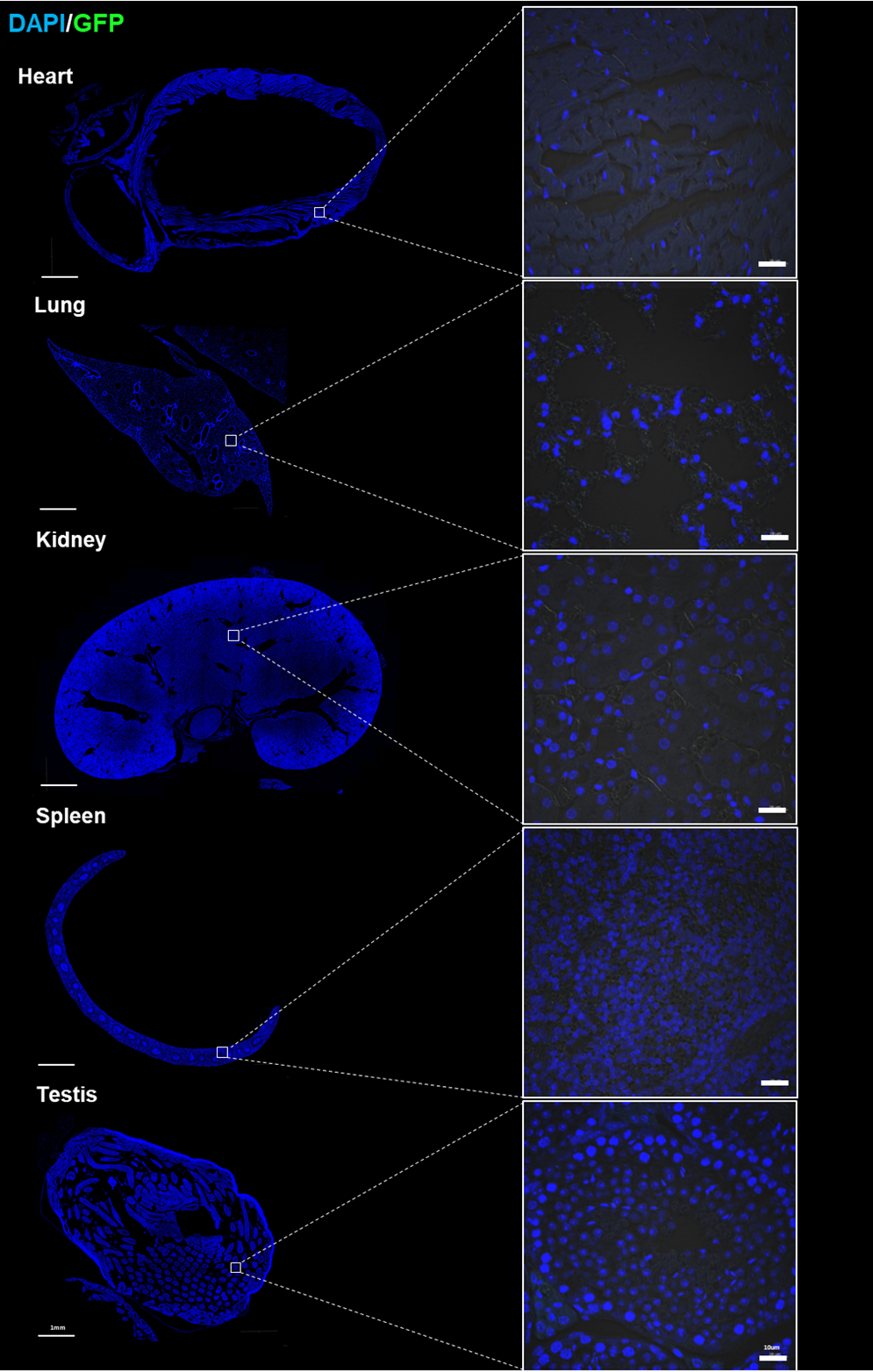


Figure S3. Immunofluorescence staining of mouse tissue. GFP staining of tissues in mouse heart, lung, kidney, spleen, and testis. LAS X software was used for visualizing the tissues under the Leica microscope. Left scale bar: 1 mm. Right scale bar: 20 µm.


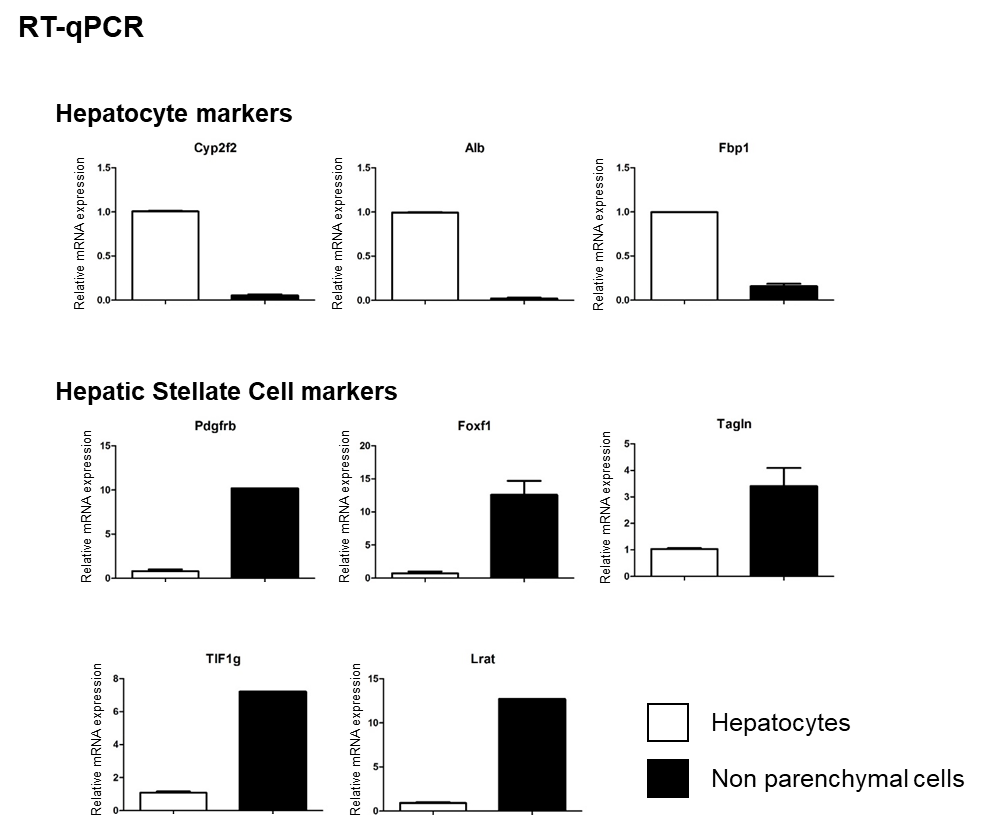


Figure S4. Reverse transcription-polymerase chain reaction (RT-qPCR) of isolated cells from mouse liver. The hepatocyte-related genes *Cyp2f2*, albumin gene, and *Fbp1* and hepatic stellate cell-related genes *Pdgfrb*, *Foxf1*, *Tagln*, *TIF1γ*, and *Lrat* were used to validate the cells. Cyp2f2: Cytochrome P450 2F2, Alb: Albumin, Fbp1: Fructose-bisphosphatase 1, Pdgfrb: Platelet-derived growth factor receptor beta, Foxf1: Forkhead box F1, Tagln: Transgelin, TIF1γ: Transcriptional intermediary factor 1 gamma, Lrat: Lecithin retinol acyltransferase. B2M used for normalization.


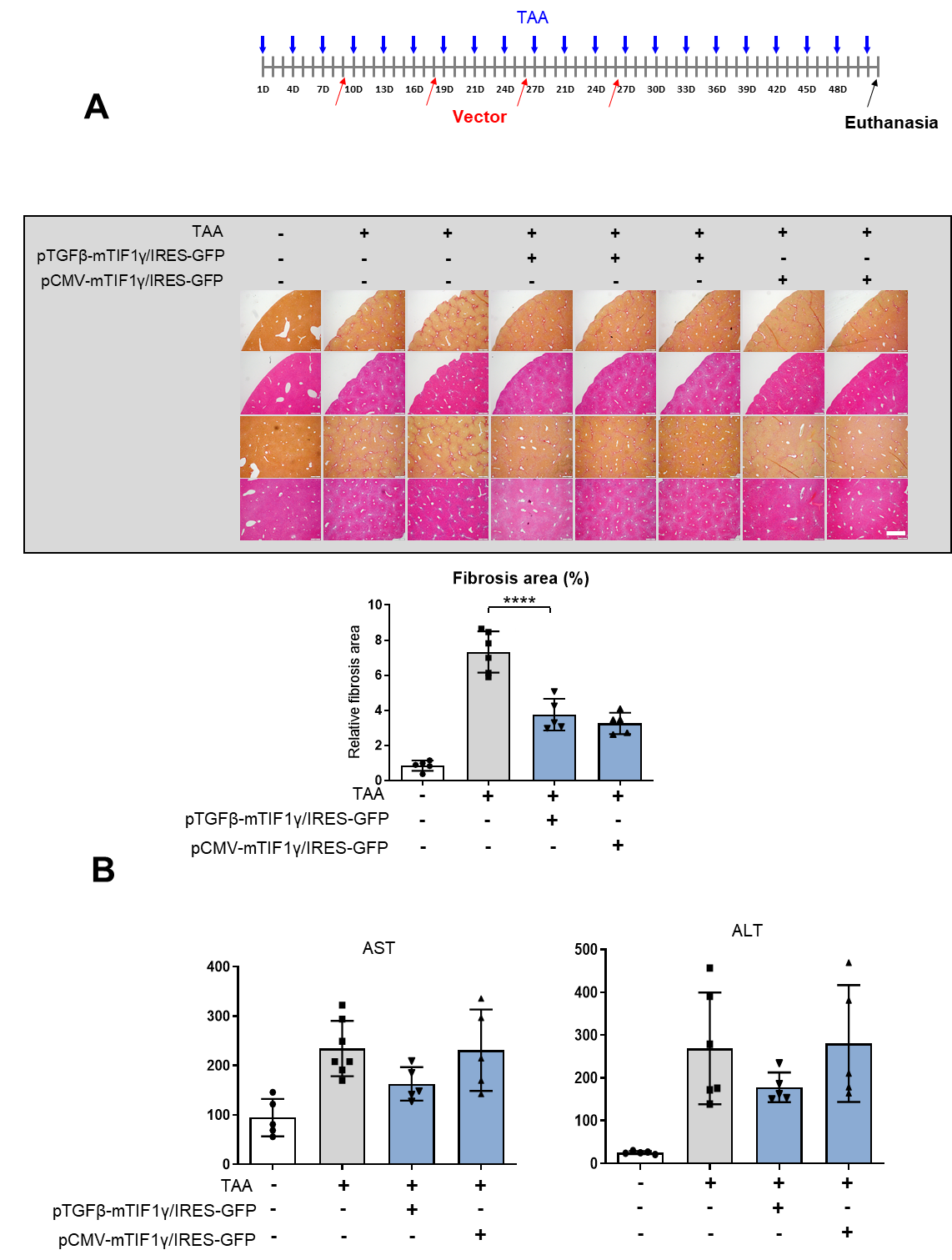


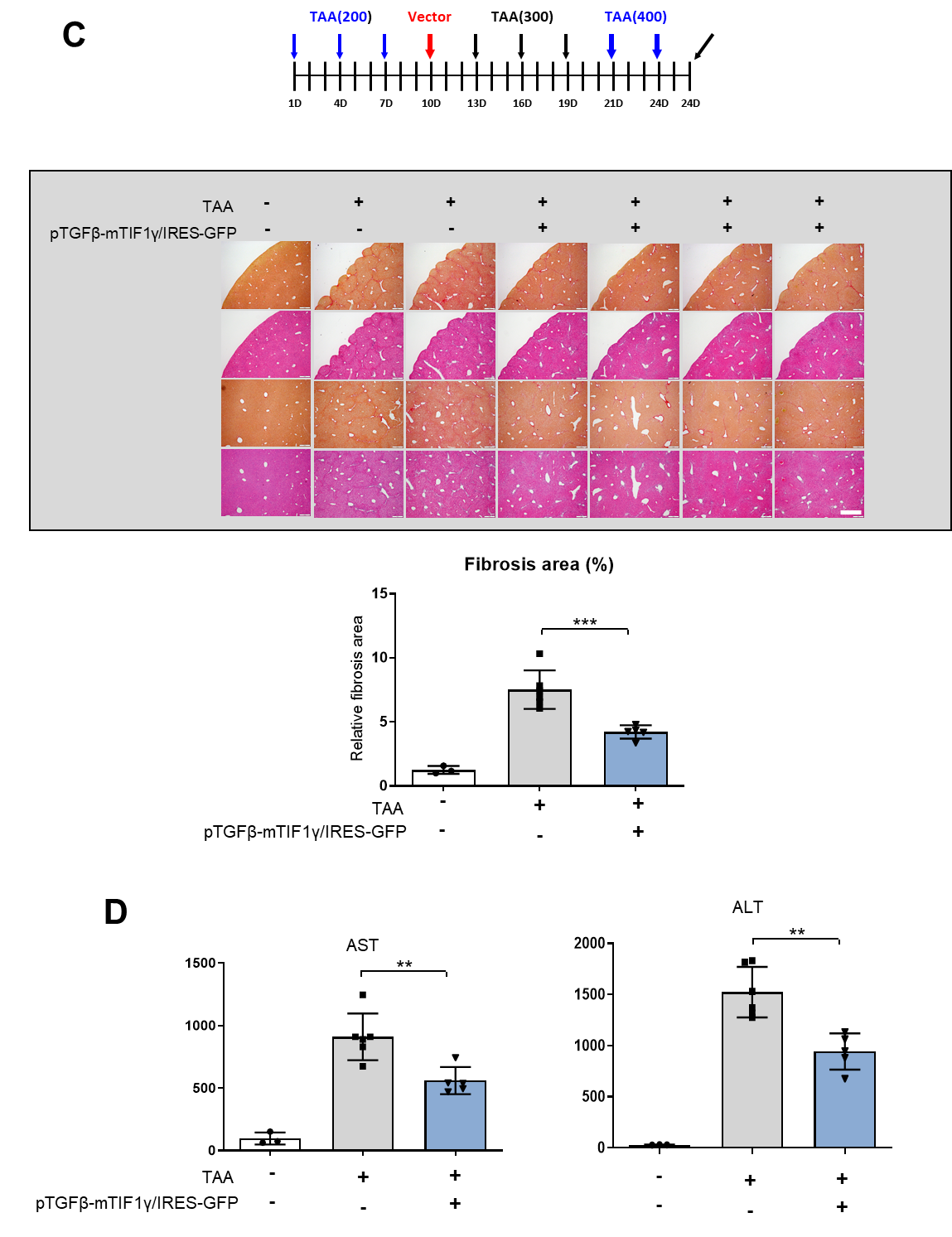


Figure S5. Effectiveness of the construct TGFβ1 promoter-driven TIF1γ packaged in LiVitA in TAA-induced mouse liver fibrosis model.

(A) Fibrosis staining and quantification in mouse liver. Experimental schema of systemic administration of the vector four times into mice with liver injury induced by TAA administration. Quantification of liver fibrosis using Picro-Sirius Red staining in four groups (normal, TAA, pTGFβ1-mTIF1γ/IRES-emGFP, and pCMV-mTIF1γ/IRES-emGFP plasmid). Each black pattern in the graph indicates an independent individual mouse. Mice, n ≥ 5 in each group. Quantification of the fibrotic area is presented as the red portion (%) in the total area. Scale bar: 400 µm.

(B) Aspartate aminotransferase (AST) and alanine aminotransferase (ALT) levels in the mouse serum. Each black pattern indicates an independent individual mouse. Mice, n ≥ 5 in each group.

(C) Fibrosis staining and quantification in mouse liver. Experimental schema of systemic injection of vector once into mice with TAA-induced liver injury. Quantification of liver fibrosis using Picro-Sirius Red staining in three groups (normal (mice, n = 3), TAA (n = 5), and pTGFβ1-mTIF1γ/IRES-emGFP plasmid (n = 5)). Each black pattern indicates an independent individual mouse. Quantification of the fibrotic area is presented as the red portion (%) in the total area. Scale bar: 400 µm.

(D) Serum AST and ALT levels were reduced by pTGFβ1-mTIF1γ plasmid in mice. Each black pattern indicates an independent individual mouse.


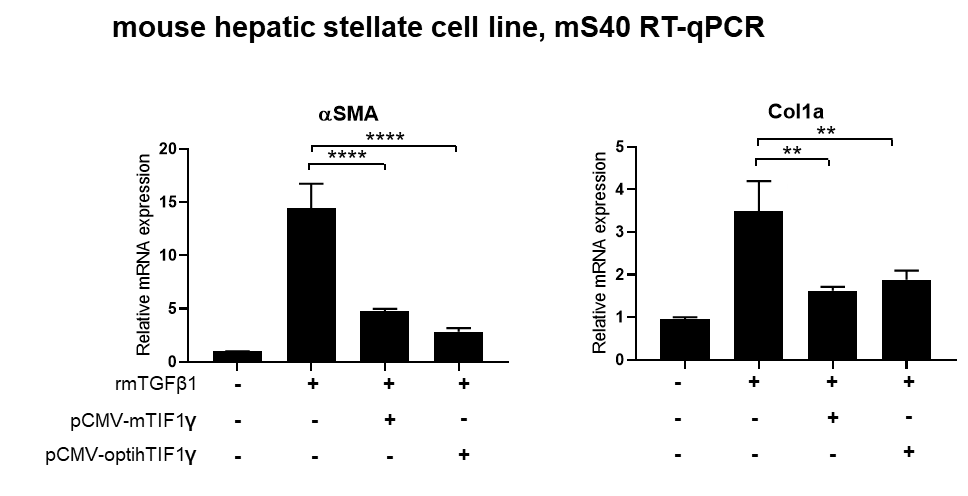


Figure S6. Reverse transcription-polymerase chain reaction (RT-qPCR) of mS40 cells. To confirm the functionality of human TIF1γ including the construct in mouse hepatic stellate cells (HSCs), pCMV-optiTIF1γ-transfected mS40 cells were used. After TGFβ1 treatment, downregulation of the fibrosis-related genes *αSMA* and *COL1A* was detected at similar levels to that for mouse TIF1γ including the construct. GAPDH used for normalization.

A
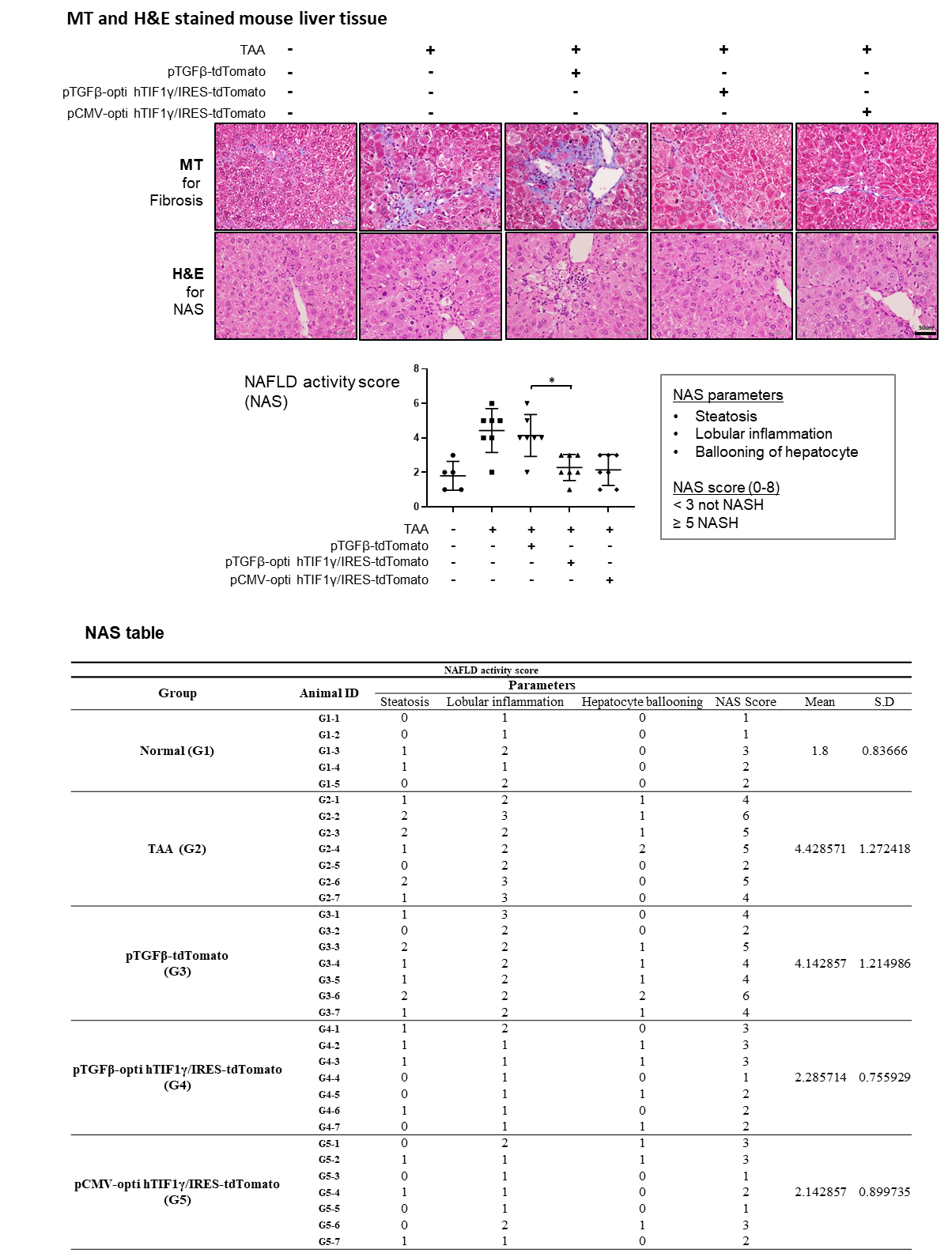


B
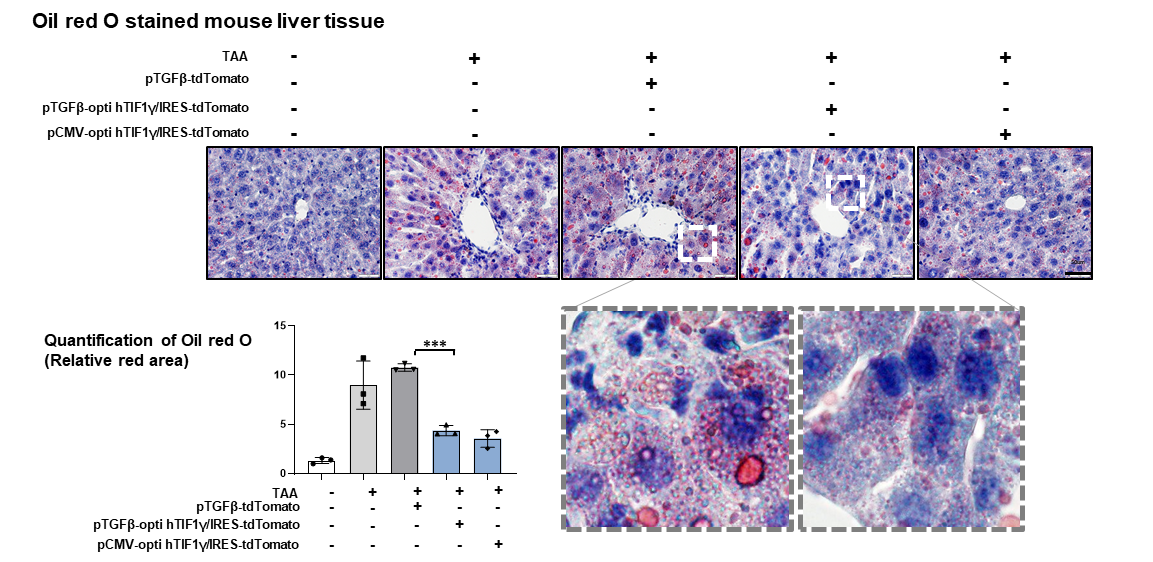


C
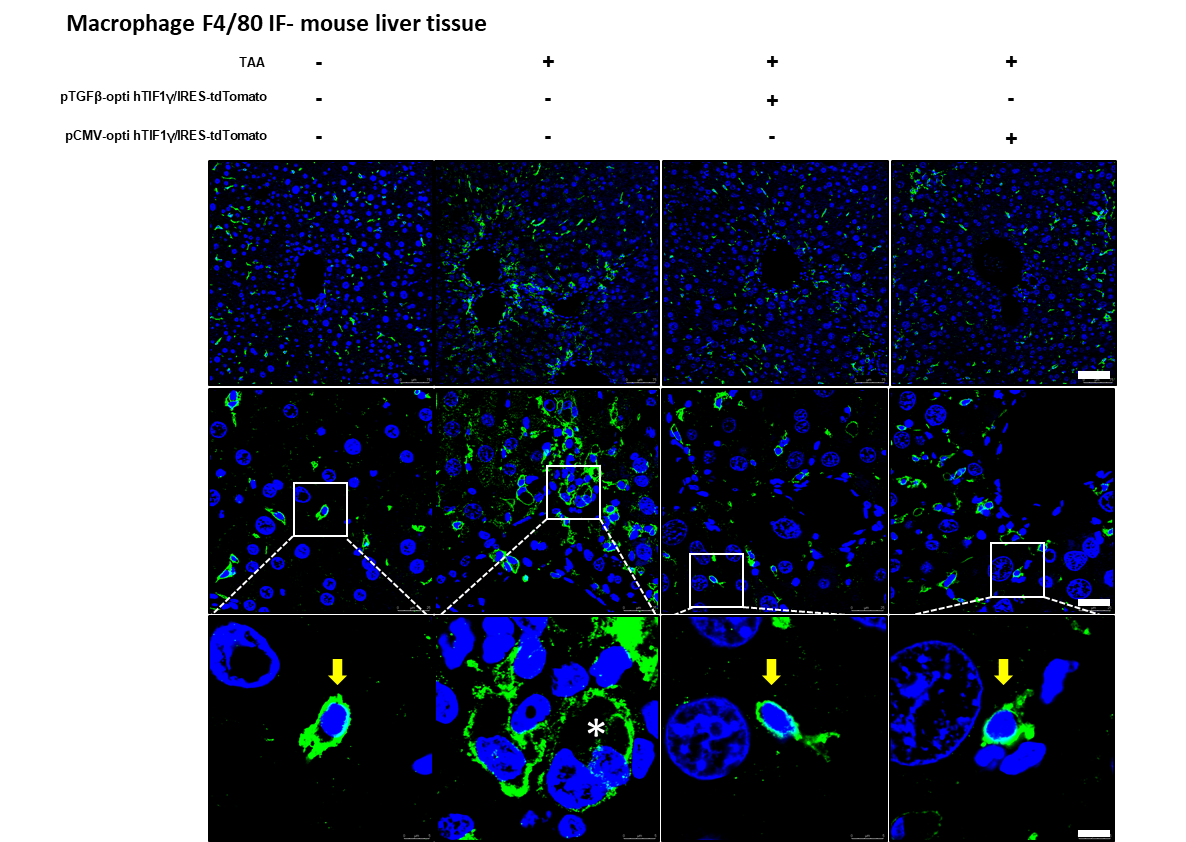


D
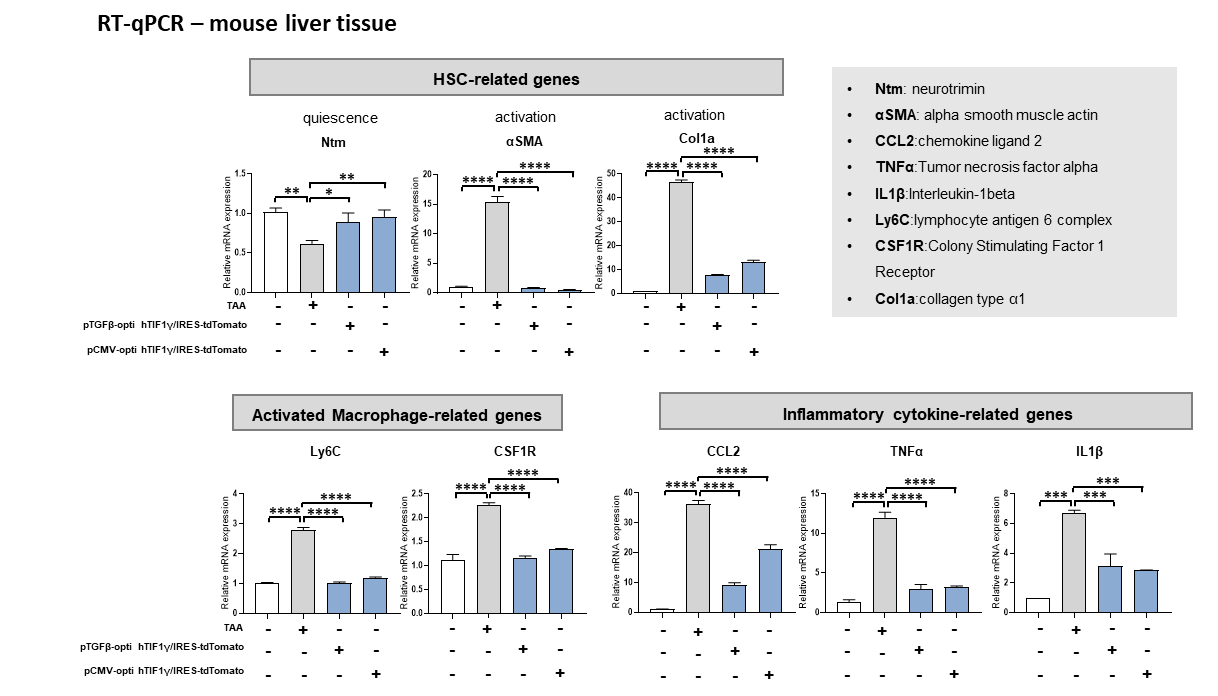


E


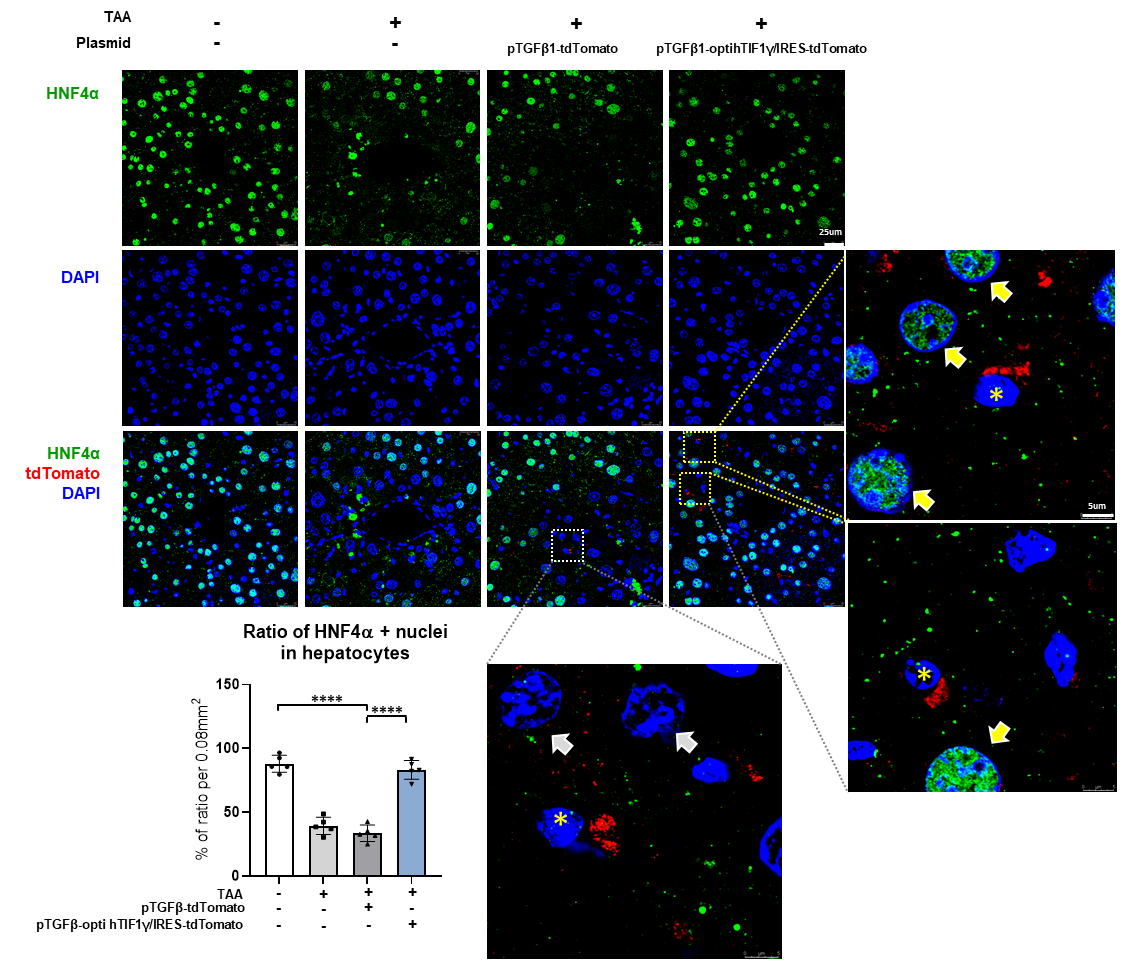


Figure S7. Quantification of histological changes in mouse liver tissue. (A) Masson’s trichrome staining and hematoxylin and eosin staining for nonalcoholic fatty liver disease activity score (NAS; steatosis, lobular inflammation, and ballooning of hepatocyte used as NAS parameters). The table showed the individual score of each NAS parameter. Scale bar: 50 µm. (B) Oil red O staining for microvesicular steatosis of hepatocyte. Scale bar: 50 µm. (C) Macrophage F4/80 immunohistostaining. White asterisk indicates foam-like phenotype macrophage with proliferation, and yellow arrows indicate inactivated macrophage. Scale bar: 75 µm in the upper panel, 25 µm in the middle panel, and 5 µm in the bottom panel. (D) RT-qPCR of mouse liver tissue. The expression of HSC quiescence-related gene *Ntm*, HSC activation-related genes (*αSMA*, *Col1A*), activated macrophage-related genes (*Ly6C*, *CSF1R*), and inflammatory cytokine-related genes (*CCL2*, *TNFα*, *IL1β*) was analyzed. B2M used for normalization. (E) HNF4α and tdTomato immunohistostaining. Yellow asterisks indicate HSCs to which the vector was delivered by LiVitA, yellow arrows indicate uninjured hepatocytes that express HNF4α+ nuclei, and white arrows indicate injured hepatocytes and loss of HNF4α in nuclei. Scale bar: 25 µm and 5 µm in the magnified panel.

**Materials and Methods**

**LX2 Culture**

The human HSC line LX2 used in this study was a generous gift from Dr. Friedman. LX2 cells in high-glucose Dulbecco’s modified Eagle medium (DMEM) supplemented with GlutaMax (Cat. 10566016; Gibco), 2% fetal bovine serum (FBS; Cat. 16000; Gibco), and 1% (v/v) penicillin/streptomycin (cat. 15070063; Gibco) (LX2 complete medium) were cultured in a humidified incubator with 5% CO^2^. The risk of mycoplasma contamination in the cells used in this study was assessed using the MycoQsearch™ Mycoplasma Real-Time PCR Detection Kit (CellSafe, Seoul, Korea). To validate the constructed vector in HSCs, the vector was transfected with Fugene HD (Cat. E2311; Promega) and 10 ng/ml of rhTGFβ1 (Cat. 7754-BH-005; R&D) was added to the culture daily for 3 or 5 days.

**mS40 Cell Culture**

Immortalized mouse HSCs were purchased from Applied Biological Materials (Cat. T0688, abm) and named mS40. mS40 cells were grown in a complete medium at 33 °C in a humidified incubator containing 5% CO_2_. The complete medium was composed of high-glucose DMEM supplemented with GlutaMAX (Cat. 10566016; Gibco), 2% FBS (Cat. 16000; Gibco), and 1% penicillin/streptomycin (Cat. 15070063; Gibco). The risk of mycoplasma contamination of the cells used in this study was assessed using the MycoQsearch™ Mycoplasma Real-Time PCR Detection Kit (CellSafe). Cell culture dishes were thinly coated with rat tail Collagen I (Cat. A1048301; Thermo Fisher Scientific). Collagen I was diluted to 50 μl/ml using 20 mM acetic acid. Cells were pretreated with recombinant mouse TGFβ1 protein (10 ng/ml; Cat. 7666-MB-005; R&D) 3 days before transfection and for one more day after transfection. The vectors (pCMV-mTIF1γ and pCMV-optimized hTIF1γ) were transfected with Fugene HD (Cat. E2311; Promega).

**HepG2 Cell Culture**

The human hepatocyte cell line HepG2 was purchased from the Korean Cell Line Bank (cat. 88065). HepG2 cells in high-glucose DMEM (Cat. 11995065; Gibco), 10% FBS (Cat. F0900-050; GenDEPOT), and 1% (v/v) penicillin/streptomycin (cat. 15070063; Gibco), i.e., HepG2 complete medium, were cultured in a humidified incubator containing 5% CO^2^. The risk of mycoplasma contamination of the cells used in this study was assessed using the MycoQsearch™ Mycoplasma Real-Time PCR Detection Kit (CellSafe). To validate the constructed vector in hepatocytes, the vector was transfected with Fugene HD (Cat. E2311; Promega) and 10 ng/ml of rhTGFβ1 (Cat. 7754-BH-005; R&D) was added to the culture daily for 3 days.

**mTIF1γ and hTIF1γ Homology Analysis**

mTIF1γ and hTIF1γ nucleotide sequence homology was analyzed using the Global Alignment BLAST tool (National Center for Biotechnology Information [NCBI]) and the Multalin Alignment tool.

**Codon-optimization of hTIF1γ**

The optimized coding sequence of hTIF1γ was obtained and synthesized using GeneArt™ GeneOptimizer™ software (Thermo Fisher Scientific). This sequence was sub-cloned into the pVAX1 plasmid downstream of the CMV promoter. The expression of the construct was assessed in 293T cells. Cell lysates were subjected to western blotting at 24, 48, and 72 h after transfection.

**Animal Model**

All animal experiments were performed and reported according to the ARRIVE (Animal Research: Reporting of In Vivo Experiments) guidelines, and all in vivo studies were approved by the Institutional Animal Care and Use Committee (IACUC no.19-0177-S1A2(1)) of Seoul National University Hospital, Korea or (BA-2001-288-008-01) CNbiologics, Korea. BALB/C and BALB/c-nude mice (weighing 20–25 g, male, 12–13-week-old) were used for the experiments. To induce liver fibrosis, TAA was injected into the mice at doses of 200, 300, and 400 mg/kg (Cat. 163678, Sigma-Aldrich) through an intraperitoneal injection. Vectors (pTGFβ1-tdTomato, pCMV-emGFP, pTGFβ1-hTIF1γ, pCMV-hTIF1γ, pTGFβ1-mTIF1γ/IRES-emGFP, pCMV-mTIF1γ/IRES-emGFP, pTGFβ1-optimized hTIF1γ-/IRES-tdTomato, and pCMV-optimized hTIF1γ/IRES-tdTomato) were injected into the mice (18 µg/head) through an intra-cardiac injection or tail-vein injection.

**Preparation of Liposome-Vitamin-A Conjugates Containing Plasmid**

*Liposome-Vitamin-A (LiVitA) Conjugation*

A mixture of 280 nmol retinol (Cat. R7632, Sigma-Aldrich) and 1 mM LipoTrust (Cat. CSR-LEO-10-EX, CosmoBio) was left for 5 min at room temperature (RT, 26 °C).

*Packaging Plasmid in LiVitA*

LiVitA (140 nmol) and plasmid (18 µg) were vortexed briefly and incubated for 20 min at 25 ℃. The solution was transferred to a Vivaspin2 concentrator into a PES column (Cat. VS0221, VIVASIENCE) and centrifuged three times at 1,500 *g* for 5 min at 25 ℃. Then the upper part of the column was reversed and its contents were centrifuged at 3,000 *g* for 2 min at 25 ℃. Next, 100 µl of 5% sucrose solution was added to reach the appropriate volume necessary for carrying out the study.

**Antibodies**

Rabbit polyclonal a-TIF1γ (Cat. ab84455, Abcam), rabbit polyclonal a-TIF1γ (Cat. ab47062, Abcam), mouse monoclonal a-αSMA (Cat. A5228; Sigma-Aldrich), rabbit polyclonal a-αSMA (Cat. ab5694, Abcam), mouse monoclonal a-αSMA-660 (Cat. 50-9760-82, Thermo Fisher Scientific), rabbit polyclonal a-Collagen 1 (Cat. PA5-29569, Thermo Fisher Scientific), mouse monoclonal β-actin (Cat. sc-47778, Santa Cruz Biotechnology), mouse monoclonal a-GAPDH (Cat. ma5-15738, Thermo Fisher Scientific), mouse monoclonal a-TGFβ1 (Cat. sc-130348, Santa Cruz Biotechnology), rabbit polyclonal TGFβ1 (Cat. ab92486, Abcam), rabbit polyclonal a-GFP (Cat. A6455, Thermo Fisher Scientific), rabbit polyclonal a-GFP-488 (Cat. A21311, Thermo Fisher Scientific), mouse monoclonal a-CRBP1 (Cat. sc-271208, Santa Cruz Biotechnology), rabbit polyclonal a-CRBP1 (Cat. sc-30106, Santa Cruz Biotechnology), rabbit a-F4/80 (Cat# 30325T, Cell Signaling Technology), goat polyclonal a-tdTomato (Cat. AB8181-200, OriGene), mouse monoclonal a-PCNA (Cat. MA5-11358, Thermo Fisher Scientific), rabbit monoclonal a-HNF4α (Cat. Ab181604, Abcam), and mouse monoclonal a-Ki67 (Cat. 9449S, Cell Signaling Technology) were used for immunoblot and immunofluorescence assays.

**Transmission Electron Microscopy and Zeta Potential Analyses**

For these analyses, 1 mM LipoTrust, LiVitA, and LiVitA + GFP were prepared to analyze the characteristics of LipoVitA. The corresponding procedures are the same as those described for the preparation of the vitamin A-conjugated liposome with plasmid, excluding the vivaspin2 step. Morphological examination was performed using a transmission electron microscopy (TEM) system (JEM-1400, JEOL). Briefly, 1 µl of each sample was added to each TEM grid and dried for 5 min. The excess solution was then removed by absorbing the excess with a filter paper before the analysis. We then determined the particle size and zeta potential using a dynamic light scattering detector (ZetaView, Particle Metrix). Briefly, additional DPBS was added to each sample to make up a total volume of 1 ml. Next, particle size and zeta potential were analyzed using the ZetaView program.

**Immunohistochemistry**

Mouse liver tissues were perfused with cold phosphate-buffered saline (PBS) and removed. The liver tissues were fixed in 4% paraformaldehyde (PFA; Cat. 163-20145, Wako) solution, embedded in paraffin, and cut into serial sections (4–5-µm thick). Paraffin sections (mouse liver and human liver) were stained with Masson’s trichrome stain and Picro-Sirius Red stain (Cat. 365548, Sigma-Aldrich) using standard protocols. Picro-Sirius Red staining was performed to detect collagen in connective tissues.

Images were obtained using a Leica light microscope (Leica, Wetzlar, Germany). To evaluate the therapeutic effect of the vector on liver fibrosis, the percentage of the fibrotic liver area was estimated through the quantitative image analysis of Picro-Sirius Red-stained sections using ImageJ software.

**Oil red O staining**

The mouse liver on OCT is sectioned into 12 µm for oil red O staining. OCT sections were stained with Oil red O staining kit (Cat# ab150678, Abcam) according to the manufacturer’s instructions. Images were obtained using a Leica light microscope. To evaluate lipid deposits in the liver, red spot (lipid) area was measured by performing a quantitative image analysis of Oil red O-stained sections using ImageJ software.

**Non-alcoholic fatty liver disease (NAFLD) activity score (NAS) analysis**

Slides with hematoxylin and eosin-stained mouse liver sections were evaluated twice in a blinded manner by a hepatopathologist. Steatosis, inflammation, and ballooning of hepatocyte were used as NAS parameters. The NAS included a numerical score for steatosis (0–3), ballooning of hepatocyte (0–2), and lobular inflammation (0–3). The sum of these scores represents the NAS. A score greater or equal to 5 correlated with a NASH diagnosis and scores of less than 3 were correlated with absence of NASH.

**Immunofluorescence analysis**

LX2 cells plated on a confocal dish (Cat. 81156, ibidi) were washed with PBS and fixed with 4% PFA (Cat. 163-20145, Wako) for 15 min. After washing with PBS, blocking solution (5% normal horse serum, 0.01% TX-100 in PBS) was used to block the cells at RT for 30 min. The cells with primary antibodies were incubated overnight at 4 °C. After incubation, the cells were washed and incubated again with secondary Alexa Fluor-conjugated antibodies (Invitrogen) and 4ʹ,6-diamidino-2-phenylindole (DAPI) for 1.5 h at RT. The cells were then washed with a washing solution (0.2% NP-40 in PBS) and then with PBS.

Human liver slides (normal, cirrhosis) were provided by the Department of Pathology, Seoul National University Hospital, Korea and purchased from Superbiochips Laboratories, Cancer Research Institute Seoul National University, Korea. This study was approved by the Institutional Review Board (IRB) of Seoul National University Hospital, Korea (IRB no. 2101-023-1185).

The paraffin-embedded tissue sections (human and mouse liver) were deparaffinized with xylene and rehydrated with graded alcohol. Sections were exposed to a heat-mediated antigen retrieval buffer (Cat# ab93684, Abcam) and then blocked with 5% normal horse serum in PBS containing 0.01% Triton X100 to block non-specific binding sites. Then, the sections were incubated with primary antibodies overnight at 4 ℃. The slides were incubated with secondary Alexa Fluor-conjugated antibodies (Invitrogen) and DAPI for 1.5 h at RT after washing them. The slides were then washed with a washing solution (0.2% NP-40 in PBS) and mounted in a fluorescence mounting medium (Cat. S3023, DAKO). Images were obtained using a Leica confocal microscope (TCS SP8).

**RT-qPCR Analysis**

Total RNA was first isolated from the cultured cells with TRIzol (Cat. 15596026, Thermo Fisher Scientific) and then extracted according to the manufacturer’s instructions. cDNA was synthesized from 1 µg of RNA using Reverse Transcription Master Premix (Cat. EBT-1512, ELPIS Biotech, Daejeon, Korea). The samples with FastStart Universal SYBR Green Master (Rox) (Cat. 04 913 914 001, Roche, Basel, Switzerland) was run in an ABI PRISM-7 500 sequence detection system (Applied Biosystems). Glyceraldehyde 3-phosphate dehydrogenase (GAPDH), peptidylprolyl isomerase A (PPIA), and beta 2 microglobulin (B2M) were used as internal controls to calculate relative changes in gene expression. Primers were designed using the Primer BLAST tool (NCBI) and synthesized by Macrogen (Seoul, Korea).

The following primers were used:

hGAPDH: forward: 5′-AAGGTCGGAGTCAACGGATTT-3′, reverse: 5′-GTTCTCAGCCTTGACGGTGC-3′;

hPPIA: forward: 5′-CATAATGGCACTGGTGGCAAG-3′, reverse: 5′-GCCATCCAACCACTCAGTCTT-3′;

hαSMA: forward: 5′-GGCAAGTGATCACCATCGGA-3′, reverse: 5′-TCTCCTTCTGCATTCGGTCG-3′;

hTIF1γ: forward: 5′-CTCCGGGATCATCAGGTTTA-3′, reverse: 5′-TCAACATGCAAGCACTCCTC-3′;

hCol1A: forward: 5′-CTGCCGTGACCTCAAGATGT-3′,

reverse: 5′-CCGAACCAGACATGCCTCTT-3′;

mGAPDH: forward: 5′- GAGTGTTTCCTCGTCCCGTAGA-3′,

reverse: 5′- AATGAAGGGGTCGTTGATGG-3′;

mB2M: forward: 5′-GTATGCTATCCAGAAAACCCCTC-3′, reverse: 5′-GTTCTTCAGCATTTGGATTTCAAT-3′;

mNtm: forward: 5′-GGACTAGCTTGAGAGCAAC-3′, reverse: 5′-CACCGGCACTCCTTGGAA-3′;

mαSMA: forward: 5′-ATCTTTCATTGGGATGGAGTCAG-3′, reverse: 5′-CTGTCAGCAATGCCTGGGTA -3′;

mCol1A: forward: 5′-GATGGTGCCAAGGGTGATACT-3′, reverse: 5′-GGAGAACCATCAGCACCTTTG -3′;

mLy6C: forward: 5′-GGACTTTGGACTGGCTAGGG-3′, reverse: 5′-TGCAGTCCCTGAGCTCTTTC-3′;

mCSF1R: forward: 5′-GGACTTTGGACTGGCTAGGG-3′, reverse: 5′-TAGGGGTTCAGACCAAGCGA-3′;

mCCL2: forward: 5′-ACCTGCTGCTACTCATTCACC-3′, reverse: 5′-TGAGCTTGGTGACAAAAACTAC-3′;

mTNFα: forward: 5′-GGTCCCCAAAGGGATGAGAAGT-3′, reverse: 5′-TTGCTACGACGTGGGCTACA-3′;

mIL1β: forward: 5′-TGCCACCTTTTGACAGTGATG-3′, reverse: 5′-ATGAGTGATACTGCCTGCCTG-3′;

mPPIA: forward: 5′-AGACAAAGTTCCAAAGACAGC -3ʹ, reverse: 5′-CGTAGATGGACCTGCCGC-3′;

mTGFβ1: forward: 5′- GCAACAACGCCATCTATGAGAA -3′, reverse: 5′-CACATGTTGCTCCACACTTGATT-3′.

**Flow Cytometry**

*In Situ Digestion*

In situ digestion was performed according to the protocol reported by Mederacke et al.^15^ Briefly, mice were sequentially perfused with EGTA, pronase, and collagenase solutions through the inferior vena cava. The digested liver was minced in pronase/collagenase solution and incubated at 40 °C in a heat block at 400 rpm for 25 min.

*Isolation of Non-Parenchymal Cells (NPCs) and Hepatocytes*

After filtering through a 100-µm strainer, the sample was centrifuged at 100 *g* for 3 min at 4 °C. The supernatant was composed of NPCs, and the pellet comprised hepatocytes. Next, the supernatant was poured into a new 50-ml conical tube which was then filled with Gey’s balanced salt solution (GBSS) to a total volume of 40 ml after resuspension. The entire solution was centrifuged at 100 *g* for 3 min at 4 ℃, the supernatant was poured into a new 50-ml conical tube, and the cell pellet was resuspended in FACS buffer (0.5% BSA, 2% FBS in PBS) and placed on ice.

Primary hepatocytes and NPCs were isolated into single cells at flow cytometric analysis and incubated with the a-GFP-488 (Cat. A21311, Thermo Fisher Scientific) antibody. Flow cytometric analysis were performed using BD FACS AriaIII (Becton Dickinson).

**Genomic (g) DNA-PCR Analysis**

gDNA was extracted from the mouse liver tissue according to a general protocol using the G-spin Total DNA Extraction Kit (Cat. 17045, Intronbio). PCR was carried out using 100 ng of the gDNA template and 0.25 µM of primers in the Accupower PCR premix (Cat. K-2016, Bioneer). PCR samples were loaded onto 1% agarose gel for electrophoresis, and images were obtained using a Gel Doc XR+ system (Bio-Rad).

The following primers were used:

Human optiTIF1γ: forward: 5′-GACCTGAGCACCGTCAAGAA-3′, reverse: 5′-AATCTCTTGTGTGTCGGCGT-3′;

tdTOMATO: forward: 5′-CAACATGGCCGTCATCAAAGA-3′, reverse: 5′-CTTGTACAGCTCGTCCATGCC-3′; GFP: forward: 5′-ATGAAGGGTGTGGACGACTG-3′, reverse: 5′-CGCACGTACATCTTCTCGGT-3′;

Internal control: forward: 5′-CTAGGCCACAGAATTGAAAGATCT-3′, reverse: 5′-GTAGGTGGAAATTCTAGCATCATCC-3′;

**Western Blot Analysis**

Total protein was extracted from cells using RIPA cell lysis buffer (Cat. 89901, Thermo Fisher Scientific) supplemented with complete protease inhibitor cocktail (Cat. P3100-001, genDEPOT) and phosphatase inhibitor cocktail (Cat. P3200-001, genDEPOT). The lysates were centrifuged at 13,000 rpm and 4 °C for 10 min after incubating them on ice for 15 min. The protein concentration was determined using a BCA assay (Cat. 23223, Thermo Fisher Scientific). Using 10% sodium dodecyl sulfate-polyacrylamide gel electrophoresis, the proteins were separated. The gel was then transferred onto a nitrocellulose membrane using an iblot 2 gel transfer device (Cat. IB21001, Thermo Fisher Scientific). The membrane was incubated with primary antibodies overnight at 4 ℃ after blocking it with 5% normal horse serum in PBS. The membrane was then washed with the primary antibodies and incubated with horseradish peroxidase-conjugated secondary antibodies. Secondary antibodies were washed out after incubation. The immunoreactive bands were detected using an ECL HRP chemiluminescent substrate. Proteins from the western blot were quantified using ImageJ.

**Serum Assay**

Mouse blood samples were collected from the hearts of anesthetized mice. Serum was separated by centrifugation at 3,000 rpm for 15 min and stored at –80 °C until analysis. To assess liver function after TAA treatment, ALT and AST activities were measured using an automatic chemistry analyzer (Hitachi 7070) according to the manufacturer’s instructions. AST and ALT activities were determined following the procedure recommended by the manufacturer and expressed as mU/ml.

**Statistical Analysis**

Statistical analysis was performed with GraphPad Prism 8 software (GraphPad Software, La Jolla, CA, USA). Data are presented as the mean ± standard deviation. Differences between groups were analyzed using one-way analysis of variance (ANOVA) with Tukey post hoc test or Kruskal-Wallis one-way ANOVA with Dunn’s post hoc test. *P < 0.05, **P < 0.01, ***P < 0.00 1, and ****P < 0.0001 were considered to be statistically significant.
